# A Comparative Study Between the Early and Late Enteral Nutrition After Gastrointestinal Anastomosis Operations

**DOI:** 10.7759/cureus.52686

**Published:** 2024-01-21

**Authors:** Asif Imran, Muhammad Ismail, Abbas Ali Raza, Tamjeed Gul, Aurangzeb Khan, Saad Ali Shah

**Affiliations:** 1 Surgery, Bacha Khan Medical College, Mardan, PAK; 2 General Surgery, Bacha Khan Medical College, Mardan, PAK

**Keywords:** hospital stay, surgery, gastrointestinal anastomosis, enteral nutrition, late feeding, early feeding, feeding strategy

## Abstract

Introduction: Intestinal anastomosis is a surgical procedure crucial for restoring the integrity of the digestive system and finds widespread application in addressing diverse gastrointestinal disorders such as tumors, inflammatory conditions, and traumatic injuries. The timing of restarting feeding after the surgery is a debated topic due to its potential impact on patient recovery. Early enteral feeding, administered soon after surgery, aims to counteract the negative effects of prolonged fasting and improve outcomes.

Objective: This study analyzed the early and late enteral feeding following gastrointestinal anastomosis surgery.

Methods: Forty patients undergoing abdominal surgery were prospectively randomized into early or late feeding groups. Demographics, laboratory values, operative time, blood loss, transfusion rates, nasogastric tube (NGT) removal, hospital stay, gastrointestinal recovery, postoperative body mass index (BMI), and complications were compared. Data was organized in Excel and analyzed using the Statistical Package for the Social Sciences (IBM SPSS Statistics for Windows, IBM Corp., Version 27.0, Armonk, NY). Qualitative data were presented with numbers and percentages, while parametric quantitative data used means, standard deviations, and ranges. Non-parametric quantitative data were represented with medians and interquartile ranges. Chi-square tests were used for comparing two qualitative groups with predicted counts less than 5, while independent t-tests and Mann-Whitney tests were employed for comparing two quantitative groups with parametric and non-parametric distributions, respectively. The analysis used a 95% confidence interval, a 5% margin of error, and considered P values less than 0.05 as significant.

Results: Early feeding was associated with significantly shorter NGT removal times (p=0.005) and hospital stays (p=0.001) than late feeding. Postprandial potassium levels were higher in the early group (p=0.007), while CRP levels were significantly lower (p=0.004). No significant differences were found in operative time, blood loss, transfusion rates, gastrointestinal recovery, postoperative BMI, or complication rates between groups.

Conclusions: Early enteral feeding appears safe and effective after gastrointestinal anastomosis surgery, potentially reducing hospital stay and improving inflammatory markers without increasing adverse events.

## Introduction

Intestinal anastomosis, a surgical procedure crucial for restoring the integrity of the digestive system, finds widespread application in addressing diverse gastrointestinal disorders such as tumors, inflammatory conditions, and traumatic injuries [1,2]. The timing of enteral feeding initiation following surgery has emerged as a point of considerable debate and interest within the medical community due to its potential impact on patient recovery, morbidity, and mortality rates [3,4].

The strategic implementation of early enteral nutrition, administered shortly after surgery, serves as a proactive measure to counteract the adverse effects of prolonged fasting [5]. This approach aims to address issues like muscle loss, immunosuppression, and delayed wound healing by supplying essential nutrients early in the postoperative phase [5,6].

The deliberate delay of late enteral nourishment is designed to mitigate specific concerns, such as anastomotic leaks or infections, ensuring the meticulous healing of the gastrointestinal system [7,8]. Postoperative malnutrition, a frequent complication, arises from factors like extended fasting, vomiting, and diarrhea, further compromising the immune system and escalating the risk of infection [9,10].

This comparative research focuses on assessing the advantages and disadvantages of early versus late enteral feeding in gastrointestinal anastomosis surgeries, aiming to provide evidence-based insights for healthcare practitioners. The study delves into various factors, including postoperative complications, hospital stay duration, nutritional status, and overall patient well-being, with the goal of offering guidance for clinical decision-making and enhancing patient outcomes. By conducting a comprehensive examination of enteral nutrition timing intricacies, the research seeks to contribute valuable information to the medical community, empowering professionals to make informed decisions, optimize patient care, and improve postoperative interventions in this critical surgical realm. The overarching aspiration is to make a meaningful impact on the standard of care for patients undergoing gastrointestinal anastomosis surgeries.

Objective

The objective of this study is the analysis of early versus late enteral feeding in gastrointestinal anastomosis operations.

## Materials and methods

Study design

The study, conducted at Bacha Khan Medical College (BKMC) in Mardan, Pakistan, from March to August 2023, is a comparative investigation comparing the outcomes of early versus late enteral nutrition after gastrointestinal anastomosis operations. The research aims to provide insights into the optimal postoperative care for patients undergoing these surgeries.

Inclusion and exclusion criteria

Patients with gastrointestinal anastomosis procedures aged 17 to 55 years, male or female, were included in the research. The following were included in the exclusion criteria: Patients who were younger than 17 years old; those who had co-morbid conditions like renal, hepatic, diabetes, hypertensive, or cardiac issues; people with autoimmune diseases; patients who had undergone revisional anastomosis surgery; patients who presented with adhesive intestinal obstruction; patients who had significant peritoneal cavity contamination prior to surgery; patients who had pre-anastomotic diversion (such as colostomy, gastrostomy, or enterostomy); patients who had received neoadjuvant chemotherapy, antituberculous medications; or patients who were vitally unstable or polytraumatized with accompanying spinal fractures were all excluded from the study.

Sampling techniques

Forty patients were included in the current study and were split into two groups for the study: Early postoperative enteral feeding was started in group A either 24 hours after the procedure or right away after the nasogastric tube (NGT) was removed. Depending on each patient's tolerance, the feeding was gradually increased to 100 milliliters per hour from the starting rate of around 50 milliliters per hour. Patients who responded well to this treatment plan went on to drink clear juice and then a semi-solid meal. For those who could not handle the early feeding, oral fluid intake was stopped for 12 hours, and then feeding was resumed at a reduced speed.

Enteral feeding in group B was administered according to standard protocol, starting as soon as patients demonstrated the return of intestinal sounds, the absence of distention in the abdomen, and the passing of either stool or flatus.

An assessment was conducted on both groups to compare the results of early and late enteral feeding. Clinical factors included in this evaluation included the time at which audible intestinal sounds were heard, the passage of flatus or stool, the occurrence of symptoms such as nausea, vomiting, or distention in the abdomen, the need to re-install the NGT, surgical wound infections, anastomotic leaks, and the length of hospital stay. Complete blood counts, serum albumin levels, and measures of potassium, sodium, and other pertinent indicators were among the laboratory evaluations. An extensive examination of the effects of early vs late enteral feeding was provided by carefully comparing each of these parameters between the two groups.

Statistical analysis

The data was initially organized using Excel and then imported into the Statistical Package for the Social Sciences (IBM SPSS Statistics for Windows, IBM Corp., Version 27.0, Armonk, NY) for analysis. Qualitative data were presented using numbers and percentages, while parametric quantitative data were described using means, standard deviations, and ranges. Non-parametric quantitative data were represented using medians and interquartile ranges (IQR). In comparing two qualitative groups with predicted counts in a cell less than 5, the Chi-square test was used. For the comparison of two quantitative groups with parametric and non-parametric distributions, independent t-tests and Mann-Whitney tests were utilized, respectively. The statistical analysis adopted a 95% confidence interval with a 5% margin of error, and P values less than 0.05 were considered significant.

Ethical statement

The research, involving 40 participants who underwent elective or emergency small- or large-intestine anastomose surgery, received ethical approval from the Institutional Review Board (IRB) of Bacha Khan Medical College Mardan, Pakistan (reference no. 142/BKMC dated: 08/02/2023). Adhering to the ethical guidelines of the Helsinki Declaration, written informed consent was obtained from all participants before their inclusion. Gastrointestinal procedures were performed using the surgeon's preferred methods, and antibiotics were prescribed based on each patient's pre- and post-operative medical status.

## Results

This study comprised 40 individuals who underwent various abdominal surgical procedures. Table 1 demonstrates that there were no statistically significant differences between the early and late feeding groups in terms of demographic characteristics such as gender, age, smoking status, and preoperative body mass index.

**Table 1:** Comparison of Variables Between Early and Late Feeding Groups SD: standard deviation, P-value: <0.05 statistical significance

Variables		Early feeding		Late feeding		Chi-square test	
		Patient number (n)	Percentage (%)	Patient number (n)	Percentage (%)	X2	P value
Age	Mean ± SD	47.50±10.01		42.75±12.73		1.124	0.271
Early Body Mass Index	Mean ± SD	30.29±5.03		31.00±6.00		-0.350	0.729
Gender	Female	10	25	10	25	0	1
	Male	10	25	10	25		
Consumption of Smoking	Yes	5	25	9	45	1.429	0.232
	No	15	75	11	55		

The comparison of laboratory data between the early and late feeding groups is shown in Table 2, which also includes the mean values, standard deviations, and the results of an independent t-test. With the exception of potassium (K), bilirubin total, and bilirubin direct levels, which showed significant variations across the groups, the majority of the variables showed no significant changes.

**Table 2:** Comparison of Laboratory Values Between Early and Late Feeding Groups Early/Late Feeding: Treatment groups in the study, SD: standard deviation, T: Test statistic for comparing means between groups, P-value: <0.05 statistical significance, WBC: White blood cell count, RBG: Random blood glucose, INR: International normalized ratio, Na: Sodium, K: Potassium

Variables	Early feeding	Late feeding	Independent t-test	
	Mean±SD	Mean±SD	T	P value
Hemoglobin	12±1.3	12.25±1.13	-0.565	0.577
WBC	12.5±2.93	12.06±4.63	0.304	0.763
Platelets	250.71±61.15	246.88±63.3	0.168	0.868
Albumin	3.79±0.43	3.5±0.52	1.638	0.113
RBG	159.43±34.94	164.31±39.61	-0.356	0.725
INR	1±0	1±0	-	-
Na	138.5±3.92	138.44±2.71	0.051	0.959
K	3.93±0.27	3.51±0.51	3.219	0.003
Bilirubin total	0.58±0.18	0.88±0.29	-3.349	0.002
Bilirubin direct	0.21±0.06	0.32±0.1	-3.313	0.003

The "Early feeding" and "Late feeding" groups' operative hours are contrasted in Table 3. The "Late feeding" group (103.13) had a significantly higher mean operative time than the "Early feeding" group (101.71). With a p-value of 0.866, the t-test, however, reveals a non-significant difference, suggesting that this discrepancy is probably the result of chance.

**Table 3:** Comparison of Operative Time Between Early and Late Feeding Groups SD: standard deviation, P-value: <0.05 statistical significance

Variables	Early feeding	Late feeding	Independent t-test	
	Mean±SD	Mean±SD	T	P value
Operative time	101.71±19.02	103.13±25.43	-0.170	0.866

Table 4 compares blood loss and transfusion rates between patients who received early or late feeding after surgery. While 35% of early feeders and 20% of late feeders received blood transfusions, the difference was not statistically significant. Early feeders experienced slightly higher mean blood loss (288.57 mL) compared to late feeders (263.75 mL), but this difference was also not statistically significant. These findings suggest that the timing of feeding after surgery may not significantly affect blood loss or transfusion requirements.

**Table 4:** Comparison of Blood Transfusion and Blood Loss Between Early and Late Feeding Groups SD: standard deviation, P-value: <0.05 statistical significance

Variables		Early feeding		Late feeding		Chi-square test	
		Patient number (n)	Percentage (%)	Patient number (n)	Percentage (%)	X^2^	P value
Blood transfusion	Yes	7	35	4	20	0.403	0.526
	No	13	65	16	80		
Blood loss	Mean ± SD	288.57±84.66		263.75±78.05		0.835	0.411

Table 5 shows statistically significant variations in the length of hospital stay and NGT removal between the early feeding and late feeding groups. The initial idea was to remove the NGT the day after surgery for the early feeding group. To relieve their symptoms, four patients had vomiting and distension of the stomach, necessitating the reinsertion of the NGT. If there were no further episodes of vomiting or distension in the abdomen, the NGT would then be reopened and feeding would resume. Patients who received early feeding notably spent less time in the hospital than those who received late feeding.

**Table 5:** Comparison of Variables Between Early and Late Feeding Groups With Independent t-test Analysis SD: standard deviation, P-value: <0.05 statistical significance, NGT: Nasogastric tube

Variables	Early feeding	Late feeding	Independent t-test	
	Mean±SD	Mean±SD	T	P value
Nasogastric tube amount	385.00±156.34	507.81±177.87	-1.995	0.056
NGT removal day	2.50±0.52	3.25±0.77	-3.067	0.005
Hospitalization duration	5.71±1.73	7.94±1.24	-4.089	0.001

Table 6 reveals no significant differences in gastrointestinal recovery between early and late feeders. The average time for the first intestinal sounds was 2.14 and 2.56 hours in the early and late groups, respectively, with no statistically significant difference (p=0.182). Similarly, the average time for passing gas or stool was slightly longer in the late group (3.06 hours) compared to the early group (2.57 hours), but this difference didn't reach statistical significance (p=0.071). This suggests that the timing of feeding does not noticeably affect the return of gut sounds or bowel movements after surgery.

**Table 6:** Comparison of Gastrointestinal Recovery Times Between Early and Late Feeding Groups With Independent t-test Analysis SD: standard deviation, P-value: <0.05 statistical significance

Variables	Early feeding	Late feeding	Independent t test	
	Mean±SD	Mean±SD	T	P value
Time of presence of intestinal sounds	2.14±0.86	2.56±0.81	-1.369	0.182
Time of passage flatus or stool	2.57±0.65	3.06±0.77	-1.873	0.071

Table 7 presents a comparison of postoperative BMI between the early and late feeding groups. It reveals that the mean BMI for individuals who started feeding early (30.96 ± 5.35 kg/m²) was comparable to those who began feeding later (30.91 ± 6.02 kg/m²). This close resemblance is further reinforced by the Chi-square test's p-value of 0.981, which indicates no statistically significant difference in BMI between the two groups. It suggests that the timing of feeding after surgery does not seem to have a noticeable impact on an individual's postoperative BMI.

**Table 7:** Comparison of Postoperative BMI Between Early and Late Feeding Groups Using Chi-Square Test BMI: body mass index, SD: standard deviation, P-value: <0.05 statistical significance

Variables	Early feeding	Late feeding	Chi-square test	
			X^2^	P value
Postoperative BMI Mean±SD	30.96±5.35	30.91±6.02	0.024	0.981

The relationship between early and late eating patterns and several laboratory indicators is examined in Table 8. For every marker, the means, standard deviations, and p-values are shown. Interestingly, there were no significant changes seen in most indicators; nevertheless, a notable rise in potassium levels was related to early feeding.

**Table 8:** Association of Feeding Style With Laboratory Markers Early/Late Feeding: Treatment groups in the study, SD: standard deviation, P-value: <0.05 statistical significance, Hbg: Hemoglobin, WBC: White blood cell count, Na: Sodium, K: Potassium

Variables	Early feeding	Late feeding	P value
	Mean±SD	Mean±SD	
Hgb	11.6±0.9	11.6±0.7	0.866
Wbc	14.6±1.7	14.3±2.0	0.869
Platelets	240±66	242±73	0.631
Na	139±3	139±3	0.627
K	3.9±0.3	3.4±0.3	0.007
Albumin	3.9±0.2	3.5±0.3	0.000

A comparison of post-cranial pressure levels and patient distribution between the early and late feeding groups is shown in Table 9. Compared to late feeding, early feeding was linked to considerably lower CRP levels (less than 10 mg/dl) (p-value = 0.004).

**Table 9:** Comparison of Post CRP Levels and Patient Distribution in Early and Late Feeding Groups Using Chi-Square Test CRP: C-reactive protein, mg/dl: milligrams per deciliter, P-value: <0.05 statistical significance

Variables		Patient number (n)	Percentage (%)	Patient number (n)	Percentage (%)	X^2^	P value
Post CRP (mg/dl)	<10	13	65	8	40	2.625	0.004
	>10	7	35	12	60		

Together with the findings of Chi-square tests for the three clinical symptoms of fever, vomiting, and abdominal distension, Table 10 displays the patient distribution and matching percentages for the early and late feeding groups. The results of the Chi-square test (X2) and P values in the table show that there is no statistically significant correlation between the time of meal and these symptoms.

**Table 10:** Association Between Early vs. Late Feeding and Clinical Symptoms in Patients P-value: <0.05 statistical significance

Variables	Early feeding		Late feeding		Chi-square test	
	Patient number (n)	Percentage (%)	Patient number (n)	Percentage (%)	X^2^	P value
Fever						
Yes	11	55	14	70	1.071	0.301
No	9	45	6	30		
Vomiting						
Yes	10	50	13	65	0.475	0.491
No	10	50	7	35		
Abdominal distension						
Yes	6	30	9	45	0.741	0.389
No	14	70	11	55		

Data on the incidence of three postoperative complications - anastomotic leaking, surgical site infection, and ICU need - for patients in the early and late feeding groups are shown in Table 11. The non-significant P values corroborate the Chi-square test findings, which show that there is no statistically significant difference in the incidence of these problems between the two feeding groups.

**Table 11:** Comparative Analysis of Anastomotic Leakage, Surgical Site Infection, and ICU Requirement in the Investigated Group P-value: <0.05 statistical significance

Variables	Early feeding		Late feeding		Chi-square test	
	Patient number (n)	Percentage (%)	Patient number (n)	Percentage (%)	X^2^	P value
Anastomotic leakage						
Yes	0	0	2	10	0.905	0.341
No	20	100	18	90		
Surgical site infection						
Yes	4	20	10	50	2.625	0.105
No	16	80	10	50		
Need for ICU						
Yes	7	35	9	45	0.201	0.654
No	13	65	11	55		

## Discussion

To assess hospital stay, recovery time, and complications after gastrointestinal anastomosis surgery, this research examined enteral nourishment administered early vs. late. A big or small intestine anastomosis was performed on 40 individuals undergoing elective or emergency abdominal surgery. The purpose of this research was to evaluate enteral feeding after gastrointestinal anastomosis, both early and late.

In this research, the postoperative BMIs of both groups were comparable. According to Hortencio et al. [11], there is no connection between malnutrition, as determined by BMI and mineral issues. BMI indicates dietary status but not recent weight loss, which is associated with mineral deficiencies. Variations in weight among hospitalized patients were mostly due to fluid balance associated with hemodynamic and inflammatory problems rather than energy balance.

In this experiment, there was no significant difference in blood loss or transfusion between early and late feeding. Due to blood loss of 288.57±84.66 and 263.75±78.05, respectively, blood transfusions were given to 35% of early feeders and 20% of late feeders. Marwah et al. [12] discovered that blood loss occurred in 68% of early eaters and 60% of late feeders (mean 242±89.52 and 284±143.41, respectively). The blood loss in both groups was statistically not significant.

NGT removal day increased statistically in this experiment. NGT removal days were 2.50 for early feeding and 3.25 for late feeding. According to Negi et al. [13], the first drink was given to the early feeding group 38.14±38.50 hours after surgery, while the late feeding group got it 50.09±51.80 hours later [13].

In this study, there was no statistically significant difference seen between early and late feeding in terms of digestive noises or stool passage. Bowel noises were heard again after 2.57 days with early feeding and 3.06 days with late feeding. In contrast, the review article [14] explores the broader context of early oral feeding (EOF) after gastrointestinal surgery, emphasizing that while most patients tolerate EOF, a notable percentage may not do so until the fourth postoperative day, with EOF offering limited advantages over delayed feeding in terms of complications. The study by Maeboud et al. [15] investigates the efficacy of postoperative gum chewing in cesarean section patients, finding that gum chewing is safe, well-tolerated, and associated with accelerated intestinal motility recovery, leading to a shorter hospital stay.

In this research, hospitalization for early feeding increased statistically significantly for 5.71 days and for late feeding for 7.94 days. Hospital stays were similarly impacted by delayed feeding [16]. Arif et al. [17] observed that hospital stay was short in the early feeding group being 19 ± 1.95 hours versus 29 ± 6.7 hours (p-value 0.03) in the delayed feeding group. A total of 5.8 days were spent in the postoperative hospital after early feeding, and 7.01 days were spent after late feeding [12]. Early feeding was observed by Negi et al. [13] to shorten hospital stays. While the late feeding group spent 71.00 ± 73.99 hours in the hospital, the early feeding group spent 52.58 ± 54.71 hours [13]. The research group's hospital stay was shortened by early NGT removal and early feeding, whereas the control group may have had greater complications and a longer hospital stay (pneumonia, upper respiratory tract infection).

A total of 35.7% of early feeders and 43.8% of late feeders in this research required ICU treatment. According to Faris et al. [18], there was no statistically significant difference seen between the two groups' mean ICU stays for enteral feeding (4.65 ±2.29 days) and parenteral nutrition (5.68 ±2.74 days) [18].

Compared to late feeding (3.50), early feeding did not increase postoperative albumin (3.9). Marwah et al. [12] likewise discovered that early eaters had significantly higher postoperative blood protein levels than late feeders, even though preoperative levels were the same. In this investigation, feeding later raised potassium by 3.40 and feeding early by 3.93. Kumar et al. [19] stated that the gastrointestinal system is prepared for abdominal surgery and anesthesia by the use of colon-cleaning agents, oral cathartics, cathartic enemas, and dietary restrictions. On the other hand, postoperative gastrointestinal function may be impacted by frequent perioperative hypokalemia [20].

Vomiting, temperature, and stomach distension did not vary substantially between early and late feeding in this experiment. Abdominal distension was 30% during early feeding and 45% during late feeding. Fifty percent of those fed early and 65% of those fed later puked. Fever from early feeding was 55%, and from late feeding, 70%. A study reported no discernible difference in vomiting or stomach distension between early and late feeding [21]. Although there was little stomach distension in both groups, 10% of the early eaters puked. Marwah et al. [12] also found no significant difference in distention rates across groups.

Early feeding postoperative CRP in this research was 35%. Enteral 10/40 (25%) patients had fewer postoperative infections than parenteral 16/40 (40%) cases, according to research by Faris et al. [18]. Early feeding reduces famine, improving nutrition, metabolism, and perioperative stress response. Transluminal endotoxemia, bacteremia, and mucosal atrophy are the results of starving the gut. In a separate study by Masood et al. [22], they conducted a randomized controlled trial to assess Enhanced Recovery After Surgery (ERAS) protocols, particularly EOF, in emergency abdominal surgeries for perforated duodenal ulcer repairs. The results showed that EOF led to significantly shorter hospital stays, lower pain scores, and reduced postoperative ileus duration, with no observed duodenal repair site leaks in the early feeding group [22].

In the current study, anastomotic leakage and surgical site infection were not substantially affected by early or late feeding. Surgical site infection occurs 20% early and 50% late in feeding, and anastomotic leaks 0% early and 10% late. Marwah et al. [12] found four (16%) early-feeders and seven (28%) late-feeders developed wound discharge. After surgery, anastomotic leaks occurred in two (8%) of the early feeders and three (12%) of the late feeders. In the current study, feeding timing did not substantially affect anastomotic leakage or surgical site infection rates, with a 20% occurrence of surgical site infection in the early feeding group and 50% in the late feeding group. Anastomotic leaks were reported in 0% of early feeders and 10% of late feeders. In contrast, Tanaka et al. [23] investigated anastomotic leakage in rectal surgery without finding statistically significant risk factors, except for a higher occurrence in male patients. However, the overall rate of anastomotic leakage in their study was low, suggesting that efforts to preserve good blood flow and prevent tension and pressure on the anastomosis during surgery may have contributed to favorable outcomes. While the studies differ in focus and methodology, both contribute valuable insights into factors influencing anastomotic leakage, emphasizing the complexity of outcomes influenced by multiple variables in surgical practice.

Limitations

The current study includes a small sample size (n=40) conducted within six months at a single medical institute, limiting generalizability. The non-uniform surgical approaches, allowing surgeons to use their preferred methods, and variations in antibiotic prescriptions based on individual patient status introduce potential confounding factors, affecting the internal validity of the study.

## Conclusions

A more favorable postoperative course results from early enteral feeding after gastrointestinal anastomosis procedures. The benefits of early feeding are highlighted by the significant decrease in the length of hospital stay as well as a reduction in postoperative infections and better potassium levels. These results highlight the potential benefits of early enteral feeding for improving patient outcomes after gastrointestinal anastomosis surgeries and support its inclusion as a helpful practice in postoperative care.
